# Improvement in Adherence to Mediterranean Diet, Cooking and Food Skills Among University Students Attending a “Teaching Kitchen” Project: Results from the S.A.P.O.R.E. Initiative

**DOI:** 10.3390/foods15020302

**Published:** 2026-01-14

**Authors:** Silvia Marconi, Daniele Nucci, Giacomo Montani, Giulia Gilberti, Monica Marullo, Luca Facciano, Chiara Passeri, Barbara Zanini

**Affiliations:** 1 Department of Clinical and Experimental Sciences, University of Brescia, 25123 Brescia, Italy; silvia.marconi@unibs.it (S.M.); giulia.gilberti@unibs.it (G.G.); monica.marullo@unibs.it (M.M.); 2 Department of Hygiene and Health Prevention, Simple Departmental Structure for Food Hygiene and Nutrition, Health Protection Agency (ATS) Brescia, 25123 Brescia, Italy; daniele.nucci@ats-brescia.it (D.N.); chiara.passeri@ats-brescia.it (C.P.); 3 PhD National Program in One Health Approaches to Infectious Diseases and Life Science Research, Department of Public Health, Experimental and Forensic Medicine, University of Pavia, 27100 Pavia, Italy; 4 Dietetics and Clinical Nutrition Service, ASST Spedali Civili of Brescia, 25123 Brescia, Italy; montanigiacomo@gmail.com; 5 Agrofood Research Hub, Department of Civil Engineering, Architecture, Land, Environment and Mathematics, University of Brescia, 25123 Brescia, Italy; luca.facciano@unibs.it

**Keywords:** cooking skills, food skills, Mediterranean diet, fibre intake, diet quality, nutrition knowledge, university students, sustainability, teaching kitchen

## Abstract

Background: The aim of the S.A.P.O.R.E. (Sustainable And Pocket friendly Options for nutritious and Responsible Eating) initiative was to offer students attending the University of Brescia a teaching kitchen programme to develop cooking and food skills (CS and FS) and promote healthy food choices. Methods: The course was structured in four weekly lessons, and both before and after, participants were asked to anonymously complete validated questionnaires to assess CS and FS, adherence to the Mediterranean Diet (MD) and dietary fibre intake. Levels of competence and satisfaction were assessed at the end of the course. Results: Twenty-eight students completed the course. Mean CS and FS scores significantly increased, from 56.1 ± 19.8 to 68.0 ± 16.5 (score range 0–98, *p* < 0.001) and from 92.7 ± 22.4 to 104.3 ± 21.0 (score range 0–133; *p* = 0.012), respectively. The MediLite score significantly increased from 9.43 ± 2.77 to 10.9 ± 2.06 (*p* = 0.006). The mean daily dietary fibre intake increased slightly from 17.8 ± 8.4 to 19.2 ± 7.1 g. More than 70% of participants correctly answered the questionnaire about acquired competence. The average cost for a single meal was EUR 1.50 ± 0.60, and the mean level of students’ satisfaction was 4.4 out of 5. Conclusions: This initiative was significantly associated with improvement in CS, FS and adherence to MD, but not in fibre intake.

## 1. Introduction

Dietary habits, food purchasing decisions and culinary skills are recognized as key elements of everyday life, with the potential to influence health outcomes and to prevent several non-communicable diseases [1]. Recent studies have shown that having greater cooking and food competences, such as preparing balanced dishes from scratch, manage cooking methods or plan meals ahead, were associated with a higher nutritional quality [2,3,4,5]. Moreover, food skills related to budgeting, label reading and resourcefulness are associated with less food and money waste, key aspects of sustainable development [6,7,8].

Traditional cooking techniques, the use of seasonal and local foods, colour variation, culinary practices and conviviality are also part of the characterizing factors of the Mediterranean Diet (MD) [9]. Moreover, the MD pattern, rich in plant-based foods, may have a better ecological footprint than other dietary habits in high-income countries, especially if compared to the Western dietary pattern [10,11]. The up-to-date version of the MD pyramid emphasizes how health promotion and sustainability could be both promoted by daily food choices [12,13]. A recent study, performed among Italian and Spanish university students, demonstrated that adherence to the MD pattern positively influences both well-being and academic performance [14].

The tradition of sharing recipes and culinary skills within families is common in many countries in the Mediterranean area, but in the last few decades this habit has been declining [15]. This change is particularly evident among young people, who are facing a fast revolution in their eating habits, driven by several marketing, cultural and social factors, with a progressive decrease in adherence to many aspects of the Mediterranean lifestyle [16].

University students, especially if they live away from home and have to manage meals by themselves, in addition to their university commitments, are particularly at risk of reducing the quality of their diet [17,18]. Convenience, costs, time constraints, limited cooking and food-related skills, limited or even wrong nutrition knowledge may drive students’ choices toward easily accessible food options: readymade meals, ultra-processed microwavable foods, home delivery and/or fast foods. These poor eating habits are characterized by high intakes of sugars, saturated fats and salt, and low consumption of fibre, proteins and several micronutrients [19,20]. Moreover, the consolidation of this eating pattern is associated with a higher risk of developing abdominal obesity and chronic diseases [21].

Teaching kitchens are well-known learning techniques to enhance nutrition knowledge and cooking and food skills and are efficient in promoting healthy eating [1]. An increasing number of studies, performed on both healthy subjects and chronic patients, have reported a significative improvement in diet quality among people following teaching kitchen programmes [22,23]. According to these encouraging data, we offered a teaching kitchen course at students attending the University of Brescia and living away from home to improve their nutrition literacy, their skills in preparing and planning meals and to optimize food budget costs.

Using a pre–post-study design, without a control group, we evaluated the effect of this initiative on cooking and food skills, adherence to the MD pattern and dietary fibre intake.

## 2. Materials and Methods

### 2.1. Study Design

This teaching kitchen initiative was conducted at the University of Brescia, Italy, in collaboration with the Public Local Health Protection Agency (ATS-Brescia) and the Hospitality Training Institute “Andrea Mantegna” of Brescia. Applying a pre- and post-study design, without a control group, we assessed changes in eating behaviour and cooking skills among university students attending the course. This basic theoretical and practical course, named S.A.P.O.R.E. (Sustainable And Pocket-friendly Options for nutritious and Responsible Eating), consisted of a four-week curriculum, in March 2025, in a teaching kitchen setting. The course programme is reported in Table 1 and covers several themes including Mediterranean Diet, Healthy Eating Plate, sustainable protein sources, basic nutrition topics, meal planning, cooking techniques, food storage and safety and budgeting strategies.

The course was delivered in a blended format, either in person at the Hospitality Training Institute or synchronously online, and lessons were conducted by a multidisciplinary team composed of researchers in the field of food science, medical doctors, dieticians, cooks and healthcare assistants. Each lesson includes a theoretical part and a practical part lasting about 60 and 90 min, respectively. The practical part of the course was designed and conducted by two chef-dieticians, who were able to combine professional cooking techniques with nutritional knowledge. The lessons of the course were scheduled once a week for four weeks, after the end of the daily university schedule, starting at 6:30 p.m. Shopping lists, necessary equipment and step-by-step recipes were shared in advance using the flipped lesson style. The ingredients were selected with a preference for seasonal products at a reasonable cost. Two dishes per lesson were prepared: one to be eaten together at the end and another one to take off in a lunch box for the next day. During meal preparing sessions, each recipe was discussed for its potential challenges in preparation, its nutritional value and its taste.

### 2.2. Participants

All students in the University of Brescia received an e-mail invitation for the S.A.P.O.R.E. course through institutional channels. Enrolment was open from 1 to 15 February 2025: 12 places in person and 30 ones remotely were available, with priority to students from outside. Interested students had to follow online instruction for enrolment and specify whether they prefer to participate in person or remotely; upon confirmation of recruitment, they had to agree a consent form and to complete the baseline surveys. Students who achieved 75% attendance and completed all final surveys received a specifically associated open badge [24].

### 2.3. Data Collection

At the beginning and at the end of the course, validated self-administrated questionnaires were provided via Google Forms (Microsoft Corporation; Washington, DC, USA).

The adherence to the Mediterranean Diet was assessed by the MediLite survey, setting the consumption of nine food groups (fruits, vegetables, cereals, legumes, fish, meat, dairy, alcohol and olive oil) and assigning a total score ranging from 0 to 18 [25,26]. Cooking and food competences were measured with a validated questionnaire, recently translated and adapted to the Italian population [3,27]. The survey was organized in two sections: the cooking skills (CS) confidence measure, consisting of 14 items, and the food skills (FS) confidence measure, consisting of 19 items; these were measured according to a seven-point Likert-scale (score 1 corresponding to “very poor ability” to score 7 corresponding to “very good ability”), with the minimum–maximum scores ranging 0–98 and 0–133, respectively (Supplementary Material Table S1). The dietary fibre intake among students was assessed whit a validated instrument (dietary fibre intake short food frequency questionnaire—DFI–FFQ), recently translated and adapted for the Italian population [28,29]. The questionnaire, structured in five separated boxes, estimates daily fibre intake by assessing consumption frequency of fruits, vegetables, bread and cereals, nuts and seeds, and legumes; according to estimated fibre level intake, subjects were classified as low (<18 g/day for females and <22 g/day for males), moderate (18–24.9 g/day for females and 22–29.9 g/day for males) or high (>25 g/day for females and >30 g/day for males) consumers.

To assess the level of acquired nutritional knowledge and practical skills, a specific questionnaire was developed consisting of fifteen multiple-choice questions based on the topics covered during both the theoretical and the practical parts of the course. The questions tested participants’ knowledge on dietary and nutrition recommendations, characteristics of raw ingredients and appropriate methods in cooking and storing foods (Supplementary Material S.2).

The overall satisfaction with the course, the level of course requirements and the learning gain were measured using a format consisting of ten questions with a 5-point Likert scale, (1 corresponding to “totally disagree” and 5 corresponding to “totally agree”) with the last open-ended question allowing students to share suggestions for future editions (Supplementary Material S.3). Free-text answers were evaluated through a lexical analysis, carried out by copying the answers in the free software wordcounter.io 2025 [30]. The software was used to calculate the frequencies of each word used in the corpus. Moreover, according to the frequency with which they recur, a word-cloud was created [31].

### 2.4. Data Analysis

Descriptive statistics were computed for the two data collection periods (before and after the S.A.P.O.R.E. course) and included frequencies and percentages for categorical variables and mean values with standard deviations for continuous variables. Further statistical analyses of the data collected before and after the course were performed for paired samples, using anonymized codes. After assessing normality (Shapiro–Wilk test) paired sample t-tests or Wilcoxon tests were used as appropriate. Statistical analysis comparing female and male populations were performed by the Mann–Whitney test for independent samples.

For all cases, the significance level was set at *p* < 0.05. Analyses were performed by Jamovi 2.6.26 open-source Software, and graph processing was performed with Microsoft Excel programme [32,33]. Given the nature of the study, a sample size calculation was not provided.

### 2.5. Ethical Considerations

This study was conducted according to the guidelines in the Declaration of Helsinki 2000, and the participants, adult students enrolled at the University of Brescia, joined the course on a voluntary basis. Before completing any questionnaire, participants read and signed an informed online consent form (Supplementary Material S.4). The questionnaires were voluntary completed, and students could withdraw their participation from the survey at any stage. To guarantee participants’ confidentiality, anonymous questionnaires were used, and all study procedures were in accordance with the provisions of the General Data Protection Regulation (GDPR 679/2016). Due to the anonymous nature of this survey, personal data could not be traced and, consequently, the protocol study did not require the approval of the local ethics committee.

## 3. Results

### 3.1. Characteristics of Participants

By 15 February 2025, a total of 216 applications had been received, 95 from off-site students. Based on the established criteria, all 42 available places were assigned; 39 students started the course (12 in person and 27 remotely); among them, 28 (8 in person and 20 remotely) attended more than 75% of the programme and completed all questionnaires. According to a per-protocol analysis, the group of students attending in person consisted of five males (62.5%) and three females (37.5%), while sixteen (80%) females and four males participated remotely. The average age of the whole sample was 25.2 ± 5.5 years old, with females aged 24.8 ± 5.5 and males aged 26.0 ± 5.7 years old. Regarding the university degree course attended, 11 (39.3%) participants were from medical, 10 (35.7%) from the engineering, 5 (17.9%) from economics and statistics and 2 (7.1%) from law.

### 3.2. Cooking Skills

At the end of the course, the average CS score increased from 56.1 ± 19.8 to 68.0 ± 16.5 (*p* < 0.001). The average score, measured on a 1–7-point Likert scale, also increased significantly from 4.01 ± 1.42 to 4.71 ± 1.18 (*p* < 0.001). CS scores increased among both females and males, from 60.0 ± 21.1 to 68.3 ± 16.5 (*p* = 0.014) and from 46.1 ± 12.4 to 61.0 ± 16.3 (*p* = 0.009), respectively. “Chop, mix and stir foods”, “Peel and chop vegetables” and “Boil or simmer food” were the skills in which students achieved the highest scores at the end of the course. The average changes in each item score are reported in Table 2.

### 3.3. Food Skills

FS scores were overall significantly improved after the course from 92.7 ± 22.4 to 104.3 ± 21.0 (out of 133; *p* = 0.012). The average score, measured on a 1–7-point Likert scale, also significantly increased from 4.88 ± 1.18 to 5.49 ± 1.11 (*p* = 0.013). FS improved among both females and males: from 99.2 ± 21.2 to 109.3 ± 18.7 (*p* = 0.051) and from 79.2 ± 19.6 to 93.7 ± 22.8 (*p* = 0.064), respectively. Before the course, but not after, FS scores were significantly different between males and females (*p* = 0.009). Following the course, the participants demonstrated an overall improvement in food skills usage and confidence, with an increase in the number of used food skills from 4 to 16. Among food competences, “Read the best-before date”, “Compare prices before you buy food” and “Shop with specific meals in mind” reached the highest scores at the end of the course. Details of FS items and scores are reported in Table 3.

### 3.4. Adherence to Mediterranean Diet

**Figure 1:**
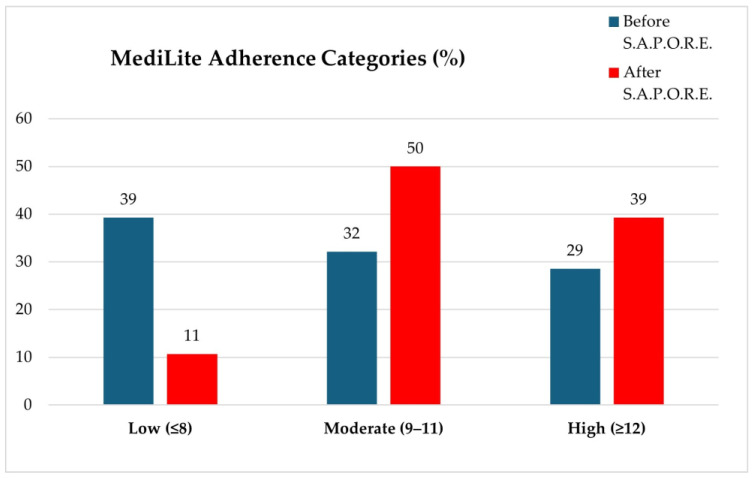
MediLite questionnaire: % of students showing low (if total score ≤ 8), moderate (if total score 9-11 and high (if total score ≥ 12) adherence, before and after S.A.P.O.R.E. course.

The average MediLite score increased from 9.43 ± 2.77 to 10.9 ± 2.06 (*p* = 0.006). Among females and males, scores increased from 10.1 ± 2.4 to 11.2 ± 1.7 (*p* = 0.06) and from 8.0 ± 3.0 to 10.2 ± 2.6 (*p* = 0.04), respectively. According to MediLite adherence categories, the number of subjects with low adherence decreased from 39% to 11% (Figure 1).

**Figure 2:**
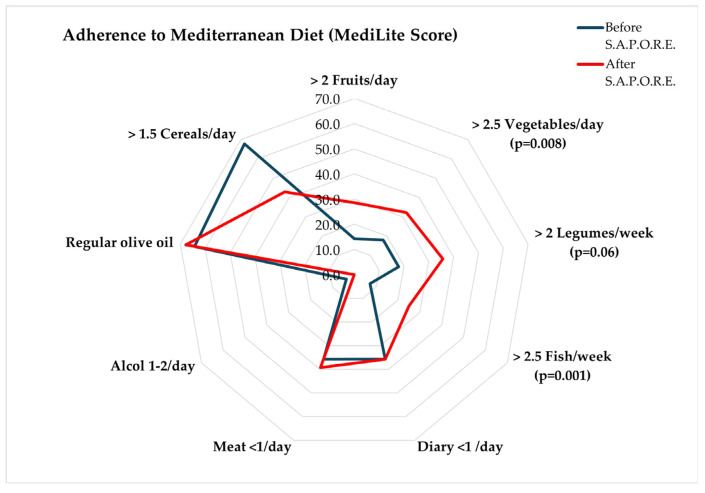
Rate of optimal adherence for fruits, vegetables, legumes, fish, diary, meat, alcohol, cereals and olive oil.

Distribution according to MediLite optimal adherence to items are reported in Figure 2. Improvement in the consumption of vegetables (*p* = 0.008) and fish (*p* = 0.001) reached a statistically significant level.

### 3.5. Dietary Fibre Intake

No significative improvement in the whole estimated daily fibre intake was reported. The mean dietary fibre intake was 17.8 ± 8.4 g/day before the course and 19.2 ± 7.1 g/day after the course (*p* = 0.236), with a median at the two time points of 17.4 g/day and 21.0 g/day (*p* = 0.218), respectively. The most consumed dietary sources of fibre were vegetables, cereals and fruit, both before and after the course, while the intake of fibre from legumes and nuts/seeds remained lower than 3 g/day and 2 g/day, respectively. A comparison of single items, before and after the course, showed a significant increase in the intake of dietary fibre only from vegetables, rising from 4.8 ± 3.6 to 6.2 ± 3.5 g/day (*p* = 0.027).

**Figure 3:**
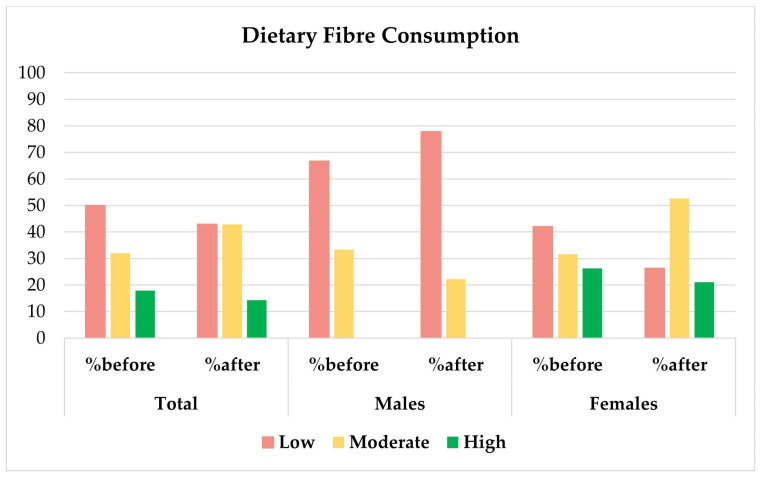
Percentage in the total sample, males and females of low (<22 g/day for males and <18 g/day for females), moderate (22–29.9 g/day for males and 18–24.9 g/day for females) and high fibre consumption (>30 g/day for males and >25 g/day for females), before and after the S.A.P.O.R.E. course.

In Figure 3, changes in the whole sample and among females and males, according to the classification into low, moderate and high fibre consumers [24,25], are reported.

### 3.6. Assessment of the Learning Level

The assessment of nutrition knowledge acquired at the end of the course showed that, overall, the rate of correct answers was above 70% for all questions, except for the first one, about the correct level for daily hydration (Supplementary Material Figure S1). Among students who attended the course in person, the rate of correct answers reached 90.0 ± 10.8%, in comparison to 81.3 ± 16.4% among students who participated remotely (*p* = 0.005).

### 3.7. S.A.P.O.R.E. Course Satisfaction Rating and Costs

About course satisfaction, an average score of 4.4 was recorded, with a minimum-maximum of 4.4–4.8 out of 5. The highest scores were recorded among questions exploring the possibility of recommending the course to other students and about acquired information about the Mediterranean Diet and its benefits (Supplementary Material Figure S2).

**Figure 4:**
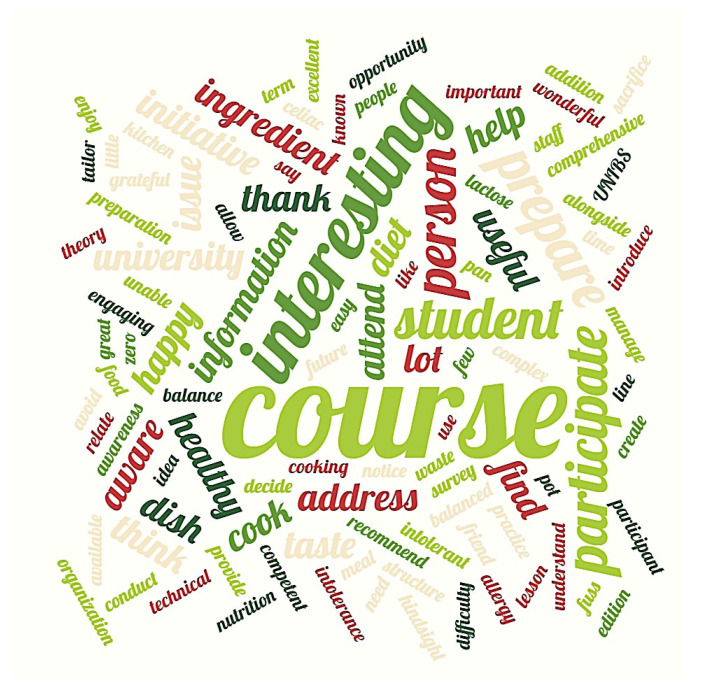
According to the frequency recurrence in the open-ended response, the word cloud showed the most frequent content words in the corpus.

A total of 9 out of 28 students added comments and/or suggestions at the end of the satisfaction questionnaire (all answers are provided in the Supplementary Material S.5). The lexical analysis of the texts listed a total of 338 words, among which the most recurrent were “course” (“corso” in Italian) 4%; “interesting” (“interessante”). These results are shown in the word cloud in Figure 4.

Lastly, we perform a cost estimation of each meal: the average cost for a single portion of each cooked dish was EUR 1.50 ± 0.60, ranging from EUR 0.30 to 2.37.

## 4. Discussion

The aim of the S.A.P.O.R.E. initiative was to offer the opportunity to improve cooking and food skills, together with nutrition knowledge, to off-site university students. This course aimed to engage students and provide them with tools to support their daily food choices, meal management and preparation, thereby promoting their health, physical and cognitive performances [31].

Food choices and eating behaviour are influenced by several factors, including social and emotional elements [34]. Encouraging university students to adopt a healthier diet based on simple, affordable and sustainable foods could promote health at the individual, community and environment levels. In this pilot project, we provided off-site students at the University of Brescia with a four-session theoretical and practical course, specifically designed to enhance participants’ knowledge and skills in meal management and preparation while keeping costs low. All participants showed a general significant improvement in both cooking and food skills, as well as in eating habits expressed as adherence to the Mediterranean Diet (especially for fish and vegetables). We also observed a slight increase in daily fibre intake, especially among females, although this remains widely below the recommended level. The course achieved excellent results regarding nutrition knowledge and competence, especially among students who attended in person. Finally, the level of satisfaction reported was very high, supporting the proposal for future editions.

In recent years, several projects aimed to reduce barriers to healthy eating by improving cooking and food skills among university students [20,35,36]. In our pilot project, the significant improvement in both meal preparation and food purchasing and management was quantified using validated tools. Furthermore, these tools allow us to identify specific skills that improved and those that need further improvement.

Comparing our results with those obtained with the same questionnaires in a cross-sectional study among 322 young sports athletes in Ireland, we observed that the participants’ mean cooking skills were 62.7 ± 17.4, quite similar to our baseline values (56.1 ± 19.8), while the mean food skill confidence was 83.8 ± 20.1, lower than that recorded by our students before and after the course (92.7 ± 22.4 and 104.3 ± 21.0) [37]. In a recent cross-sectional study aimed to characterize cooking skills among 1203 university students in the northeast of Brazil, using a different instrument, participants recording a high and a low level of cooking skills were 63.6% and 0.8%, respectively. Not learning alone how to cook and the availability and accessibility of fruits and vegetables were positively associated with a high level of CS [38]. Although significant improvement was achieved in our pilot study, these results highlight the importance of designing other group interventions to further improve the overall skill level of the students’ population.

Several studies revealed the positive association between CS and FS, dietary quality and overall health outcomes. The gradual loss of these competencies, in relation to social changes, economic issues and time constraints, led to increasing consumption of pre-packaged meals and fast foods [39,40,41]. At the same time, adherence to the Mediterranean Diet has declined, influenced by additional factors, including purchasing, organization and preparation issues, with a gradual replacement of meals prepared from scratch, or using minimally processed food, with ready-to-eat meals, often quickly prepared and eaten in solitude [9]. This trend is particularly evident among young people and university students, leading to the risk of the disappearance of the Mediterranean Diet and lifestyle among future generations, with possible negative consequences on their health, psychosocial wellbeing and academic performance.

One of the perceived barrier to the adoption of the MD is its high cost [9]. These financial concerns could be mitigated by the promotion of legumes’ consumption, typical low-cost protein foods and of purchasing seasonal products, even directly from local farmers. The present pilot course demonstrates that choosing raw seasonal foods, carefully planning meals and cooking with leftovers may offer solutions to overcome this financial issue. Moreover, the MD has been regarded, in recent years, as a sustainable dietary pattern, mainly due to the large consumption of plant-based products, with a low environmental impact [10,42,43]. As previously observed by Rosenau N. and collaborators, learning to incorporate and prepare foods with a reduced environmental impact into daily meals, reflecting greater adherence to the Mediterranean Diet, could promote more sustainable behaviours [35]. In our pilot project, planning meals in advance, careful food purchase and conservation and learning how to reuse leftovers provided students with skills in addressing sustainability and in keeping meal costs low (with an average meal budget of EUR 1.50).

Despite strong evidence supporting its multiple health benefits, dietary fibre intake remains suboptimal in many countries [44]. Italian dietary guidelines recommend a minimum daily fibre intake of 25 g [45]. Nevertheless, the average intake among Italian adults is 17 g/day, far below from the recommended reference and similar to the level of our students [46]. The S.A.P.O.R.E. course exerted a slight positive effect on fibre intake. Through recipes based on whole grains, legumes, nuts and vegetables, participants acquired practical skills incorporate and cook fibre-rich foods, but the daily intake of participants remained below recommended levels. This trend highlights the ongoing challenge in achieving adequate fibre consumption, especially among young adults, and underscores the need for stronger public health efforts in promoting fibre-rich dietary patterns.

The level of acquisition of theoretical and practical skills in the field of nutrition, assessed using a specifically designed questionnaire, provided confirmation of the effectiveness of the S.A.P.O.R.E. course. Except for questions addressing the recommended hydration level in the MD pattern, the correct response rate exceeded 70% across all other topics. The significantly higher scores registered by students attending the course in person, compared to online participants, suggest a greater effectiveness of the “on-site” teaching kitchen. In-person education has been shown to enhance student–tutor and peer interaction, thereby fostering better engagement [47,48]. Overall, both the remote and in-person S.A.P.O.R.E. course effectively improved cooking and food skills and adherence to the Mediterranean Diet. Despite remote learning offering several advantages, such as greater flexibility, time and money saving and broader accessibility, it seems to be less effective than on-site learning, especially in a course with an important practical part. We observed that directly experiencing ingredients and tools, following and being supported by chefs and dieticians, savouring textures and flavours and, above all, socializing represented tremendous added values. This aspect was highlighted in some words of the open-ended question provided by students in the satisfaction questionnaire such as “enjoy”, “help” and “friend”. Moreover, the transition to independent living may reduce conviviality, especially among off-site students, further encouraging the gradual loss of MD identity.

Regarding participants satisfaction, all the participants provided positive feedback, reporting scores above 4 in a 5-point Likert scale. The S.A.P.O.R.E. course was perceived as improving self-efficacy in food and meals management, reducing waste and food budget. Participants also reported a better awareness of the MD pattern and the intention to implement in everyday choices what they had learned during the cooking classes.

Limitations in the S.A.P.O.R.E. course and the pre–post-study analyses must be considered. Firstly, the small sample size, mainly due to organization issues, without power calculation and a control group limited the validity and the generalizability of the results. This pilot basic course represented a first step towards further editions and served to better target programmes and operative models. Secondly, only 28 students completed the entire course, despite 42 places available, and a drop-out rate of 33% was observed both in person and remotely. This study was conducted without external funding and was provided without costs for the participants, but it was not possible to offer incentives for participation, except for the associated open badge. In future editions, attention should probably be focused on real motivation to participate and on social responsibility of enrolling in a limited-access course (that means depriving other colleagues of this opportunity). Thirdly, due to the lack of socio-economic and anthropometric/clinical data, no association analyses of these factors were provided. In future, in compliance with privacy regulations and with the approval by the local ethics committee, sensitive data could be collected. Despite the use of validated questionnaires about CS, FS, MD adherence and fibre intake, we must acknowledge that they are all self-reported and reflect perceived changes rather than objectifiable behavioural ones. Despite these perceived improvements, daily dietary fibre intake remains below the recommended threshold, and the increase in MD adherence score was small, providing important indications for better targeting future editions. Moreover, the lack of a follow-up assessment does not provide us with data on the long-term effects of the course.

Despite these limitations, this study also encompasses some strengths. The S.A.P.O.R.E. course was developed and implemented by a multidisciplinary team of healthcare professionals, and the practical part was supervised by two chef-dieticians, offering a unique combination of nutrition expertise and professional cooking techniques. The course was offered without additional costs for the students. The opportunity for participants in the remote learning modality, to cook simultaneously in their own kitchens, and to directly ask questions to teachers makes this an engaging proposition, even though the in-person mode seems to remain more effective. Considering that one of the perceived barriers to adhere to the MD pattern is its economic burden, here we provide, for the first time, several examples of balanced Mediterranean meals, really “pocket friendly” and economically sustainable, as suggested by the acronyms of S.A.P.O.R.E. Moreover, CS, FS, MD adherence and fibre intake were collected with questionnaires, which have been used for years in the literature or translated, adapted to the Italian culture and validated, providing greater reliability to the data.

## 5. Conclusions

Findings from the first edition of the S.A.P.O.R.E. course supported the hypothesis that improving cooking and food competences may positively influence diet quality among university students. The course was successful in teaching how to prepare balanced meals with a limited budget. Students could also learn how to minimize food waste through proper meal planning, the use of leftovers and safe storage. When cooking and food skills are not transmitted within the family, institutions may address this gap by including dedicated courses in their curricula, even promoting conviviality and socializing. University teaching kitchens could have great potential to improve nutritional knowledge, ameliorate students diet quality and promote healthier and more sustainable daily practices.

## Figures and Tables

**Table 1:** Weekly schedule of the S.A.P.O.R.E. teaching kitchen course.

Week	Lecture Topics	Practical Teaching
1st	• Basics of healthy eating • The Harvard Healthy Eating Plate • Macronutrient correct balance • Diet variety and portion sizes	• Kitchen safety • Equipment orientation • Knife skills • How to compose a balanced meal • How to clean fish
2nd	• Principles of the Mediterranean Diet and benefits for health and longevity • Focus on whole grains	• Cooking techniques: boiling, steaming and stir-frying • Preparation of an easy traditional Mediterranean recipe with whole grains, seasonal vegetables and extra virgin oil • How to manage poultry
3rd	• Environmental sustainability in food choices • Classification of protein sources • Focus on legumes and their nutritional value	• Promote the use of sustainable and nutritious ingredients • How to properly cook legumes • Legumes for making sweet creams
4th	• Food labels reading • Food waste reduction • How to correctly store foods • Rules for food preservation and safety	• Using the leftovers to reduce food waste • Meal prep ideas for busy days • Budgeting for meals • Basic principles of batch cooking

**Table 2:** Confidence in cooking skills in the overall population before and after the S.A.P.O.R.E. course. N and % of students who responded positively regarding the use of a skill; 0 corresponds to “never/rarely used”. Mean values and standard deviation (S.D.) of score measured on a 7-point Likert scale; *p*-value < 0.05 are expressed in bold.

Cooking Skills	Used Skills		Before		Used Skills		After		*p*-Value
	N	%	Mean	S.D.	N	%	Mean	S.D.	Before vs. After
**Cooking** **Methods**									
**1. Chop, mix and stir foods**	28	100	5.36	1.77	28	100	6.07	0.90	**0.008**
**2. Blend foods to make them smooth, like soups or sauces**	22	78.6	3.79	2.77	26	92.9	5.18	2.00	**0.003**
**3. Steam food**	13	46.4	1.89	2.77	25	89.3	4.11	2.11	**<0.001**
**4. Boil or simmer food**	26	92.9	5.75	1.95	28	100	5.89	1.37	0.774
**5. Stew food**	16	57.1	2.25	2.44	23	82.1	3.29	2.35	**0.026**
**6. Roast food in the oven**	22	78.6	3.5	2.60	26	92.9	4.61	2.22	**0.008**
**7. Fry/stir-fry food in a frying pan/wok with oil or fat**	23	82.1	3.71	2.35	24	85.7	3.93	2.40	0.296
**8. Microwave food**	26	92.9	5.64	2.34	23	82.1	4.57	2.66	0.055
**Food Preparation Techniques**									
**9. Bake goods such as cakes. buns. cupcakes**	25	89.3	4.46	2.47	25	89.3	4.86	2.34	0.180
**10. Peel and chop vegetables**	28	100	5.50	1.48	28	100	6.11	1.03	0.058
**11. Prepare and cook raw meat/poultry**	24	85.7	3.79	2.39	23	82.1	4.04	2.50	0.334
**12. Prepare and cook raw fish**	23	82.1	2.64	2.15	23	82.1	3.61	2.42	**0.011**
**13.Make sauces and gravy from scratch**	23	82.1	3.18	2.31	24	85.7	4.36	2.43	**0.025**
**14. Use herbs and spices to flavour dishes**	28	100	4.61	1.79	28	100	5.36	1.52	**0.027**

**Table 3:** Confidence in food skills in the overall population before and after the S.A.P.O.R.E. course. Used skills: N and % of students who responded positively regarding the use of a skill; 0 corresponds to “never/rarely used”. Mean values and standard deviation (S.D.) of score assigned based on a 1–7-point Likert scale; *p*-value < 0.05 are expressed in bold.

Food Skills	Used Skills		Before		Used Skills		After		*p*-Value
	N	%	Mean	S.D.	N	%	Mean	S.D.	Before vs. After
**Meal Planning and Preparing**									
**1. Plan meals ahead**	24	85.7	3.93	2.46	26	92.9	5.07	2.00	**0.002**
**2. Prepare meals in advance**	24	85.7	4.61	2.42	25	89.3	4.79	2.13	0.742
**3. Follow recipes when cooking**	27	96.4	4.86	1.88	28	100	5.61	1.20	0.052
**4. Shop with a grocery list**	25	89.3	5.39	2.44	27	96.4	5.79	1.55	0.331
**5. Shop with specific meals in mind**	27	96.4	5.21	1.93	28	100	6.00	1.47	**0.039**
**6. Plan how much food to buy**	26	92.9	4.89	2.11	28	100	5.61	1.50	0.090
**Budgeting**									
**7. Compare prices before you buy food**	28	100	5.93	1.51	28	100	6.04	1.17	0.787
**8. Know what budget you have to spend on food**	25	89.3	5.07	2.23	28	100	5.36	1.79	0.434
**9. Buy food in season to save money**	25	89.3	3.54	2.17	28	100	4.71	1.76	**0.006**
**10. Buy cheaper cuts of meat to save money**	27	96.4	4.89	2.04	28	100	5.14	1.84	0.466
**Resourcefulness**									
**11. Cook more or double recipes which can be used for another meal**	23	82.1	4.96	2.67	28	100	5.68	1.68	0.130
**12. Prepare or cook a healthy meal with only few ingredients on hand**	27	96.4	4.54	2.08	28	100	5.21	1.71	0.140
**13. Prepare or cook a meal with limited time**	27	96.4	4.86	1.88	28	100	5.64	1.28	**0.044**
**14. Use leftovers to create another meal**	25	89.3	4.36	2.25	28	100	5.00	1.89	0.055
**15. Keep basic items in your cupboard for putting meals together**	28	100	5.32	1.54	28	100	5.68	1.39	0.256
**Label reading consumer ** **awareness**									
**16. Read the best-before date on food**	28	100	6.21	1.17	28	100	6.32	1.33	0.641
**17. Read the storage and use-by information on food packets**	28	100	5.29	1.58	28	100	5.68	1.70	0.183
**18. Read the nutrition information on food labels**	26	92.9	4.68	2.13	28	100	5.57	1.89	**0.017**
**19. Balance meals based on nutrition advice on what is healthy**	26	92.9	4.21	2.10	28	100	5.39	1.69	**0.001**

## Data Availability

The original contributions presented in this study are included in the article/Supplementary Material. Further inquiries can be directed at the corresponding author.

## Supplementary Materials

The following supporting information can be downloaded at: https://www.mdpi.com/article/10.3390/foods15020302/s1, Table S1: Evaluation of cooking and food skills: validate questionnaire—English Version [3]. Supplementary Material S.2: Questions for S.A.P.O.R.E.—assessment of learning level—March 2025 edition. Supplementary Material S.3: S.A.P.O.R.E. course satisfaction rating—consisting of ten questions with 5-points Likert scale, where 1 corresponds to “totally disagree” and 5 to “totally agree”. Supplementary Material S.4: Informed Consent Statement. Supplementary Material S.5: Comments and suggestions in the open-ended response at the end of the satisfaction questionnaire. Figure S1: Assessment of learning level: for each of the fifteen questions, the percentage of correct answers for the entire population is shown (blue line), followed by a distinction between students who participated remotely (light blue dotted line) and students who participated in person (red dotted line). Figure S2: The level of satisfaction was measured using a format consisting of ten questions with a 5-point Likert scale, where 1 corresponds to “totally disagree” and 5 to “totally agree”. The complete questions are included in the Supplementary Materials (S.3).

## Abbreviations

The following abbreviations are used in this manuscript:

CS	Cooking Skills
FS	Food Skills
DFI-FFQ	Dietary Fibre Intake Short Food Frequency Questionnaire
MD	Mediterranean Diet
