# Supplementary material for: Improvement in Adherence to Mediterranean Diet, Cooking and Food Skills Among University Students Attending a “Teaching Kitchen” Project: Results from the S.A.P.O.R.E. Initiative
Source: Foods. 2026 Jan 14;15(2):302. doi: 10.3390/foods15020302 (PMC12840108; doi:10.3390/foods15020302)
Supplement: Supplementary file 1 [file foods-15-00302-s001.zip › foods-4069744-supplementary.pdf]

SUPPLEMENTARY METARIAL

Table S.1: Evaluation of cooking and food skills: validate questionnaire – English Version [3].

**<u>Cooking Skills</u>**<br>Dear Participant, on a scale of 1–7, where 1 is very poor and 7 is very good, could you rate how good you are at each skill?<br>If a skill is not used, an option of 'never/rarely do it' is available.
1. Chop, mix and stir foods, for example chopping vegetables, dicing an onion, cubing meat, mixing and stirring food together in a pot/bowl.
2. Blend foods to make them smooth, like soups or sauces' (using a whisk/blender/food processor etc.)
3. Steam food (where the food doesn't touch the water but gets cooked by the steam)
4. Boil or simmer food (cooking it in a pan of hot, boiling/bubbling water)
5. Stew food (cooking it for a long time (usually more than an hour) in a liquid or sauce at a medium heat, not boiling) e.g. beef stew
6. Roast food in the oven, for example raw meat/chicken, fish, vegetables etc.
7. Fry/stir-fry food in a frying pan/wok with oil or fat using the hob/gas rings/hot plates
8. Microwave food (not drinks/liquid) including heating ready meals.
9. Bake goods such as cakes, buns, cupcakes, scones, bread etc., using basic/raw ingredients or mixes
10. Peel and chop vegetables (including potatoes, carrots, onions, broccoli)
11. Prepare and cook raw meat/poultry
12. Prepare and cook raw fish
13. Make sauces and gravy from scratch (no ready-made jars, pastes or granules)
14. Use herbs and spices to flavour dishes
**<u>Food Skills</u>**<br>Dear Participant, on a scale of 1–7, where 1 is very poor and 7 is very good, could you rate how good you are at each skill?<br>If a skill is not used, an option of 'never/rarely do it' is available.
1…plan meals ahead? (e.g. for the day/week ahead)
2…prepare meals in advance? e.g. packed lunch, partly preparing a
meal in advance
3…follow recipes when cooking?
4…shop with a grocery list?
5…shop with specific meals in mind?
6…plan how much food to buy?
7…compare prices before you buy food?
8…know what budget you have to spend on food?
9…buy food in season to save money?
10…buy cheaper cuts of meat to save money?
11…cook more or double recipes which can be used for another meal?
12…prepare or cook a healthy meal with only few ingredients on hand?
13…prepare or cook a meal with limited time?
14…use leftovers to create another meal?
15… keep basic items in your cupboard for putting meals together?

	e.g. herbs/spices, dried/tinned goods?
	16…read the best-before date on food?
	17…read the storage and use-by information on food packets?
	18…read the nutrition information on food labels?
	19…balance meals based on nutrition advice on what is healthy?

Supplementary Material S.2 : Questions for S.A.P.O.R.E. – Assessment of learning level - March 2025 edition

1. What is the recommended level of hydration in the Mediterranean diet pyramid?
    a. 2-3 liters of water per day (including coffee, herbal teas, and light broths)
    b. 6-8 glasses of water per day (including coffee, herbal teas, and light broths)
    c. There is no recommended limit for fluid intake
    d. 1-2 liters of water only

2. Where is the best place to store tomatoes after purchase?
    a. In the refrigerator
    b. At room temperature
    c. In the freezer
    d. On the radiator

3. Can frozen vegetables be a valid alternative to fresh vegetables?
    a. Absolutely not
    b. Yes, always
    c. Only if the frozen vegetables are just vegetables as they are
    d. Only if combined with legumes

4. Should chicken be washed before cooking?
    a. Yes, always, to avoid contaminating utensils and the kitchen
    b. No, because washing it can spread bacteria
    c. No, only if it is organic
    d. Yes, but only the thighs and breast

5. Should whole grains be washed before consumption?
    a. Yes, to remove the most fibrous and indigestible part
    b. No, to keep the grain intact
    c. Yes, to remove impurities/dust
    d. No, while non-whole grains should be washed

6. Why is it advisable to add water to the oil in the pan when sautéing vegetables?
    a. To dilute the fats
    b. To make the vegetables more digestible
    c. Because as long as there is water, the oil is not at risk of "burning"
    d. Because the oil cooks better

7. How to cook whole grains?
    a. By absorbing water after bringing them to a boil in an amount equal to twice the weight of the grain, then reducing the heat to a minimum or turning it off and covering the pot with a tight-fitting lid
    b. By boiling the water and then adding the grains
    c. Steaming
    d. By sautéing them in a pan with only water, strictly without oil

8. Why is it important to eat whole grains?
    a. Because they are an important source of insoluble fiber
    b. Because they lower the glycemic index of the grain
    c. because they contain all the micronutrients of the grain intact
    d. All of the above

9. Do carrots need to be peeled?
    a. Not necessarily, just wash and scrub them well
    b. It is always better to peel them for food safety reasons
    c. Yes, if they are eaten cooked
    d. No, but only if they are eaten raw

10. What is buckwheat?
    a. A cereal
    b. A gluten-free cereal
    c. A pseudo-cereal
    d. A legume

11. What is the best way to cut onions without crying?
    a. Wear glasses
    b. Wet the onions and/or the knife
    c. Grease the cutting board with oil
    d. Add salt

12. What is the salt content threshold per 100 grams for a product to be defined as high in salt?
    a. 1 gram
    b. 0.3 grams
    c. 2 grams
    d. 5 grams

13. When cutting most foods, how should the hand not holding the knife be positioned?
    a. Flat with the palm on top of the food
    b. Fingers arched over the food with the middle finger slightly protruding forward and the thumb behind for greater stability
    c. Fingers arched over the food with the index finger protruding slightly forward and the thumb behind for greater stability
    d. Thumb and index finger holding the food and the other fingers raised

14. How are the ingredients listed on the label?
    a. In alphabetical order
    b. In ascending order by quantity
    c. in descending order by quantity contained
    d. following the order in which they are used in the recipe

15. What is the difference between the best-before date (BBD) and the expiry date?
    a. The expiry date refers to quality, while the BBD refers to food safety
    b. The expiry date is a strict deadline and refers to the safety of the food, while the BBD refers to its quality characteristics
    c. The BBD is a strict deadline, after which the food must be discarded
    d. The expiry date is an approximate indication of the shelf life of the product

Supplementary Material S.3: S.A.P.O.R.E. course satisfaction rating - consisting of ten questions with 5-points Likert Scale, where 1 corresponds to "totally disagree" and 5 to "totally agree".

1. The course improved my awareness of the importance of a healthy and balanced diet.
2. The theoretical content covered during the lessons was clear and understandable.
3. The practical activities in the kitchen were useful for acquiring practical skills.
4. I found the information about the Mediterranean diet and its benefits effective.
5. The course provided me with useful tools to improve my weekly meal planning.
6. I feel more motivated to reduce my consumption of ultra-processed foods.
7. I believe that the recipes proposed are easy to replicate on my own.
8. The course has increased my awareness of environmental sustainability in relation to food.
9. I appreciated the atmosphere of sharing and collaboration created during the meetings.
10. I would recommend this course to other students living away from home.

Supplementary Material S.4: Informed Consent Statement. All subjects involved in the project gave their informed consent according to the following text:

"Benvenuta! Benvenuto!

Il questionario S.A.P.O.R.E. è organizzato in 3 sezioni di domande riguardanti il livello di aderenza alla Dieta Mediterranea, il consumo di fibra alimentare e le competenze nella preparazione e nella gestione dei pasti.

La compilazione richiederà circa 20 minuti ed il questionario è completamente ANONIMO. I dati verranno trattati nel rispetto delle norme a tutela della privacy e non sarà assolutamente possibile ricondurre le risposte date a Colei/Colui che le ha inserite.

Si raccomanda ATTENZIONE e SINCERITA' nelle risposte e si ricorda che questo è un questionario puramente conoscitivo, non esistono risposte scorrette e non verrà assegnato nessun punteggio.

Grazie e buona compilazione!

Acconsente al trattamento in maniera anonima ed aggregata dei dati personali ai sensi del Dlgs 196 del 30 giugno 2003 e dell'art. 13 GDPR?

Si, acconsento; No, non acconsento.”

Supplementary Material S.5: Comments and suggestions in the open-ended response at the end of the satisfaction questionnaire.

**Comment and suggestions in the open-ended response**
I found the course to be very comprehensive. Perhaps, for future editions, surveys could be conducted before the lessons to decide together which dishes to prepare, so that they are in line with the tastes of most participants.<br><br>The course was very useful for understanding how to best manage your diet without sacrificing taste and also avoiding waste. I am very grateful and happy to have had the opportunity to participate. I thank all the staff and the university.<br><br>The course was very interesting and well structured, with an excellent balance between theory and practice. In hindsight, I think everyone should participate in person.<br><br>It was really interesting and useful, not only because it addressed nutrition issues, but also because it is a course tailored to the needs of university students who have difficulty preparing meals. In addition, many ideas for healthy cooking were provided, and important technical information was introduced. Although I was unable to attend in person, I was very happy to have participated. It was a really great initiative.<br><br>Use fewer pots and pans<br><br>I found this course very interesting, but I think that allowing everyone to attend in person would make it much more engaging.<br><br>I would recommend cooking and creating "zero fuss" dishes with few ingredients (balanced and healthy) that are very easy to prepare, because you don't always have a lot of time and perhaps a lot of ingredients for more complex preparations. That said, I really enjoyed the course and thank you very much for everything! See you soon :)<br><br>It would also be interesting to address issues related to food intolerances and allergies to make students more aware. I have friends who are celiac or lactose intolerant, and I often notice that there is little information available.<br><br>A wonderful initiative by UNIBS. Courses like this certainly help all students to become more aware of their diet, which unfortunately is becoming less and less known. Furthermore, cooking alongside competent people has certainly helped in terms of awareness and organization in the kitchen.

Supplementary Material Figure S1

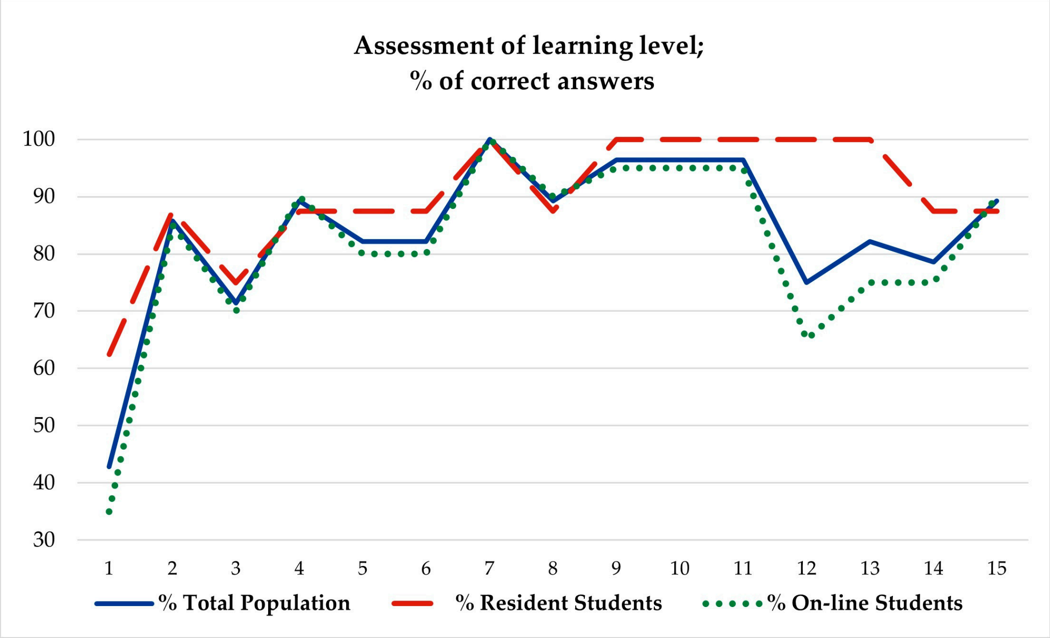

Figure S1. Assessment of learning level: for each of the fifteen questions, the percentage of correct answers for the entire population is shown (blue line), followed by a distinction between students who participated remotely (light blue dotted line) and students who participated in person (red dotted line).

Supplementary Material Figure S2

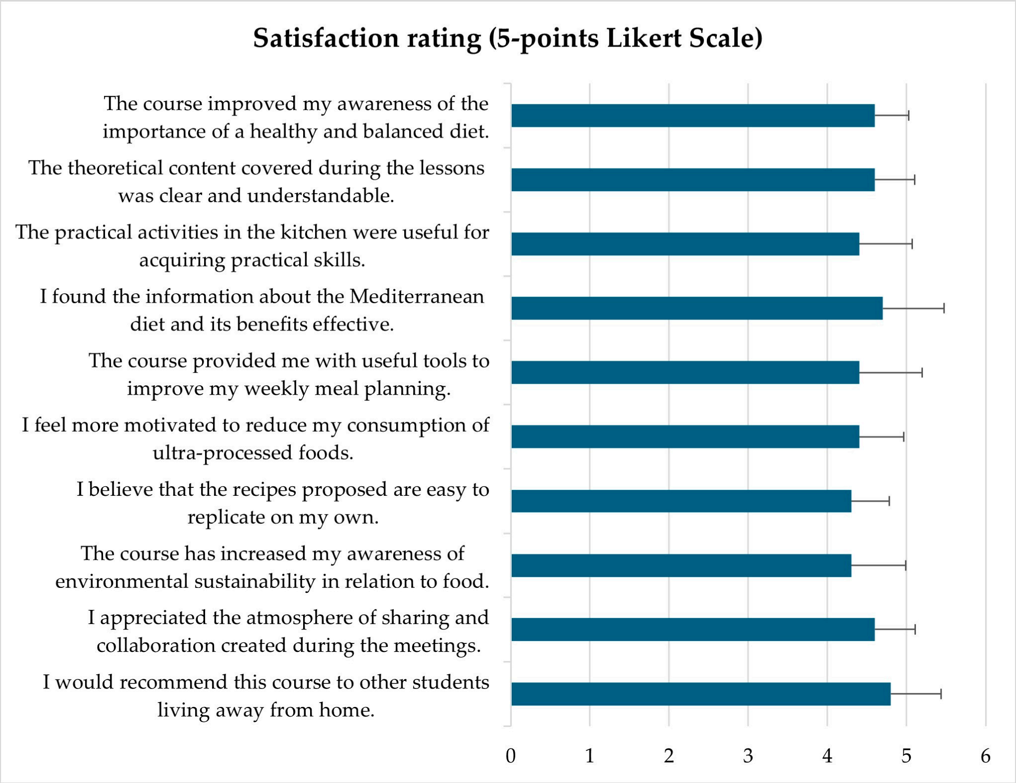

Figure S2. The level of satisfaction was measured using a format consisting of ten questions with 5-points Likert Scale, where 1 corresponds to “totally disagree” and 5 to “totally agree”. The complete questions were included in the supplementary materials (S.3).
